# Correlation Between Increase of Axial Length and Height Growth in Chinese School-Age Children

**DOI:** 10.3389/fpubh.2021.817882

**Published:** 2022-01-20

**Authors:** Lixia Tao, Chunxiao Wang, Yiyi Peng, Meiping Xu, Minghui Wan, Jiangtao Lou, Xinping Yu

**Affiliations:** ^1^Jinhua Eye Hospital, Jinhua, China; ^2^School of Ophthalmology and Optometry, Eye Hospital of Wenzhou Medical University, Wenzhou, China; ^3^State Key Laboratory of Ophthalmology, Zhongshan Ophthalmic Center of Sun Yat-sen University, Guangzhou, China

**Keywords:** axial length (AL), height, myopia, correlation, school-age children

## Abstract

**Purpose:**

To identify the relationship between the increase in axial length (AL) and height in school-age children and explore the influence of refractive status on such a relationship.

**Methods:**

In this 5-year cohort study, 414 Chinese children (237 boys) aged 6–9 years (mean 7.12) underwent measurements annually. AL was measured using the Lenstar; height with the children standing, without shoes; and refraction using subjective refraction without cycloplegia. Participants were divided according to the refractive status: persistent emmetropia, persistent myopia, and newly developed myopia. The measurement time points of the persistent emmetropia and persistent myopia groups were marked as T_1_, T_2_, T_3_, T_4_, and T_5_. The time of myopia onset in the newly developed myopia group was marked as *t*_0_; the preceding time points were marked as *t*_−1_, *t*_−2_, and so on, and the succeeding as *t*_1_, *t*_2_, and so on. The association between increase in AL and height was analyzed using simple correlation analysis.

**Results:**

The mean changes in AL, height, and refraction were 1.39 mm, 23.60 cm, and −1.69 D, respectively, over 5 years in all children. The increase in AL and height were positively correlated for T_1_~T_2_, T_1_~T_3_, T_1_~T_4_, and T_1_~T_5_ (*r* = 0.262, *P* < 0.001; *r* = 0.108, *P* = 0.034; *r* = 0.165, *P* = 0.001; *r* = 0.174, *P* = 0.001, respectively). The changes in AL and height in the newly developed myopia group were significantly correlated (*r* = 0.289, *P* = 0.009) after myopia onset (*t*_0_~*t*_2_).

**Conclusion:**

The increase in AL and height were positively correlated, especially in the newly developed myopia group after myopia onset. Thus, when children grow quickly, AL elongation should be monitored.

## Introduction

In recent decades, the prevalence of myopia has rapidly increased (1). It is predicted that nearly half of the world's population will suffer from myopia by 2050 (2). The rate may be greater for eastern Asia, including China, Japan, South Korea, and Singapore (3–5), where the incidence of myopia is higher than in other areas. School-age children are the main group of people diagnosed with myopia (6), whose elongation of axial length (AL) plays a major role in the incidence and progression of myopia (7–10). Therefore, changes in AL may reflect changes in refractive status to some extent.

The association between height and AL has been demonstrated in previous cross-sectional studies (11–14) and longitudinal cohort studies (15–17). In 2002, Saw et al. (11) proved that taller children have longer AL by analyzing the height and AL of 1,449 children aged 7–9 years. Later, in 2011, Wang et al. (15) demonstrated the correlation between them through a longitudinal cohort study. They analyzed follow-up data of 553 children aged between 7 and 15 from 2006 to 2008 and concluded that height and AL are positively correlated. In brief, all previous studies agree with the statement that height and AL are positively correlated.

However, few studies have discussed the relationship between growth in height and AL. A previous study reported that the association between height and AL is largely attributable to shared genes (18). Therefore, we predicted that an association may also be present between the speed of the growth in height and AL. Huang et al. (19) proved that average changes in height and AL were correlated in a three-year follow-up experiment. However, they did not show such association at the different stages during follow-up, and the sample size was relatively small (*N* = 88). Later, Kearney et al. (20) and Li et al. (21) also explored the correlation between the increases in AL and height, but obtained different results. Kearney et al. argued that the association existed in persistent emmetropic children, while Li et al. found no association in the entire participant cohort during the 3-year follow up. This discrepancy may be ascribed to the differences in sample size (*N* = 140 and 452, respectively) and age range (5–20 and 6–8 years old, respectively). As the elongation of AL in myopic children differs from that of emmetropic children (22–24), the AL growth of those who will become myopic accelerates before the onset of myopia and slows down after it, while the annual AL change of emmetropic children is relatively stable (22). Thus, to explore the relationship between changes in height and AL, the refractive status should be considered.

In the present study we aimed to explore the association between the changes in height and AL in children through a five-year follow-up of children aged 6 to 9 years, and to determine whether the growth in height can predict the increase in AL. Furthermore, we aimed to explore the correlation between changes in height and AL in myopic children before and after the onset of myopia.

## Methods

### Participants

This was a five-year cohort study conducted from 2015 to 2020 in Jinhua, a city situated in eastern China. The subjects were students of 10 schools in the Wucheng District, Jindong District, and Jinhua Economic and Technological Development Zone. Children with systemic diseases that affect height growth or ocular health, strabismus, or amblyopia were excluded. Participants who received myopia control treatment such as orthokeratology lenses or low-concentration atropine, other than single vision lenses, were also excluded. In total, 456 children of grades 1–4 successfully completed the baseline ocular examinations, and 414 (90.8%) continuously attended their measurements in the following examinations. The age at baseline (date of first examination) of the participants ranged from 6 to 9 years.

The study was conducted in accordance with the Declaration of Helsinki of the World Medical Association. Informed consent was obtained from all participants or their parents.

### Examinations

All participants underwent an examination at Jinhua Eye Hospital every 12 months since their first examination. The examination included height assessment and comprehensive eye examination. Height was evaluated without shoes: each child stood with the buttocks, shoulder blades, and back of the head against the wall. The doctor placed the headpiece firmly on the head and recorded the height (25). AL was measured using non-invasive, non-contact optical low-coherence reflectometry (Lenstar LS900; Haag-Streit AG, 3098 Koeniz, Switzerland) without pupil dilation. Three consecutive measurements were acquired, and the mean result was used (13). If the error of the three measurements was >0.1 mm, AL was remeasured. Refraction was measured using subjective refraction without cycloplegia by experienced optometrists. The child looked at the Standard Logarithmic Visual Acuity Chart 5 m away, while the optometrist presented a variety of lenses (including spherical lenses and cylinder lenses) and altered the power of lenses in the phoropter according to the child's subjective responses until the best-corrected visual acuity (BCVA) was achieved. The refraction was transformed into spherical equivalent (SE = sphere power + 0.5cylinder power). Refractive status was judged according to SE [myopia: SE ≤ −0.5D (26, 27); emmetropia: −0.5D < SE < +1.0D; hypertropia: SE ≥ 1.0D].

### Data Analysis

SPSS (IBM Corp. Released 2019. IBM SPSS Statistics for Windows, version 26.0. Armonk, NY: IBM Corp.) was used to analyze the data. Because data from the two eyes were highly correlated (the Spearman's rank correlation coefficient of AL and SE was 0.976 and 0.907, respectively, and both *p*-values were lower than 0.01), the data from the right eye were analyzed. The participants were classified according to the refractive status of each examination. Those who maintained emmetropia/myopia were grouped into the persistent emmetropia/persistent myopia groups. The newly developed myopia group included participants who had emmetropia or hyperopia at the first examination, became myopic in the following four examinations, and later maintained myopia. The time of each examination was marked as T_1_, T_2_, T_3_, T_4_, and T_5_ corresponding to the successive examinations for all participants/persistent emmetropia group/persistent myopia group. We then calculated the differences between the results of each examination and those at T_1_. For the newly developed myopia group, the time of first discovery of myopia was marked as *t*_0_. The previous time points were marked as *t*_−1_, *t*_−2_, …, and the following time points as *t*_1_, *t*_2_…. The difference between the results of each examination and those of *t*_0_ was calculated.

Data are presented as median (interquartile range). The correlation between the change in height and the change in AL was analyzed using simple correlation analysis. The bootstrap method was used to calculate the 95% confidence interval (95% CI) of the correlation coefficient (*r*-value). Age and sex were added as covariates in partial correlation analysis. Statistical significance was set at *P* < 0.05.

## Results

### Descriptive Data

In total, 456 children (260 boys and 196 girls) participated in this study at baseline. Forty-two children were lost to follow-up in the following four examinations and were excluded from the analysis. The remaining 414 children (57.2% boys, 42.8% girls) completed the 5-year examination cycle, including 110 in the persistent emmetropia group, 50 in the persistent myopia group, and 226 in the newly developed myopia group. Twenty-eight children had hyperopia or developed from hyperopia to emmetropia but did not develop myopia. These children were taken into account when considering the correlation in all children but not analyzed separately as a specific sub-group. There was no significant difference between participants who dropped out and the remaining participants in terms of age at baseline (*P* = 0.297), height at baseline (*P* = 0.201), AL at baseline (*P* = 0.382), or sex (*P* = 0.757). Demographic characteristics of the participants at baseline are summarized in Table 1, while the age distribution at the first examination is presented in Table 2. Height, AL, and refraction at each examination are presented in Tables 3, 4.

**Table 1:** Summary of baseline participants' demographic characteristics.

	**Children included in analysis**				**Children not included in analysis**	
	**All (*n =* 414)**	**Boys (*n =* 237)**	**Girls (*n =* 177)**	***P*** ^*^	**All (*n =* 42)**	***P*** ^**†**^
Age	7.00 (6.00 to 9.00)	7.00 (6.00 to 9.00)	7.00 (6.00 to 9.00)	0.523	7.00 (6.00 to 9.00)	0.297
H (cm)	125.00 (108.00 to 151.00)	126.00 (109.00 to 151.00)	125.00 (108.00 to 145.00)	**0.031**	128.00 (112.00 to 143.00)	0.201
AL (mm)	23.00 (20.28 to 25.17)	23.06 (20.65 to 25.17)	22.63 (20.28 to 24.92)	**<0.001**	23.00 (21.20 to 24.48)	0.382
SE (D)	0.00 (-4.13 to 9.50)	0.00 (-4.13 to 9.50)	0.00 (−3.75 to 6.25)	0.541	0.00 (−2.75 to 5.50)	**0.001**

*H, height; AL, axial length; SE, spherical equivalent*.

* *Comparison between boys and girls*.

† *Comparison between children included in analysis and those not included in analysis. Bold font indicates to point the P values < 0.05*.

**Table 2:** Age distribution of the participants in the first examination.

**Group**	**Age (years)**				**Total**
	**6**	**7**	**8**	**9**	
PE	41	44	19	6	110
PM	3	13	18	16	50
NDM	72	86	49	19	226
PH	4	4	4	0	12
Other	5	7	4	0	16
All	125	154	94	41	414

*PE, persistent emmetropia; PM, persistent myopia; NDM, newly developed myopia; PH, persistent hyperopia; Other, children who developed hypertropia into emmetropia but did not further develop into myopia during the research*.

**Table 3:** Correlation between height and AL in different groups.

**Time**	***n***	***H*** **(cm)**	**AL (mm)**	***R*** **(95% CI)**	***P***	Adj. R^*^(95% CI)	***P***
**All**							
T_1_	387	126.00 (108.00 to 151.00)	23.00 (20.28 to 25.17)	0.282 (0.180 to 0.373)	**<0.001**	0.162 (0.063 to 0.251)	**0.001**
T_2_	363	130.00 (110.00 to 159.00)	23.15 (20.53 to 25.45)	0.325 (0.224 to 0.426)	**<0.001**	0.176 (0.072 to 0.271)	**0.001**
T_3_	409	135.00 (120.00 to 168.00)	23.49 (20.87 to 25.99)	0.297 (0.202 to 0.383)	**<0.001**	0.172 (0.082 to 0.262)	**<0.001**
T_4_	405	140.50 (120.00 to 175.00)	23.85 (20.95 to 26.39)	0.297 (0.206 to 0.382)	**<0.001**	0.211 (0.112 to 0.308)	**<0.001**
T_5_	414	150.00 (120.00 to 180.00)	24.20 (21.04 to 26.62)	0.287 (0.189 to 0.378)	**<0.001**	0.245 (0.155 to 0.336)	**<0.001**
**PE**							
T_1_	92	125.00 (108.00 to 150.00)	22.81 (21.42 to 24.51)	0.318 (0.125 to 0.499)	**0.002**	0.290 (0.070 to 0.479)	**0.006**
T_2_	89	128.00 (117.00 to 153.00)	22.79 (21.00 to 24.23)	0.418 (0.225 to 0.575)	**<0.001**	0.381 (0.187 to 0.547)	**<0.001**
T_3_	109	133.00 (120.00 to 162.00)	23.11 (21.45 to 24.53)	0.293 (0.106 to 0.458)	**0.002**	0.265 (0.078 to 0.446)	**0.006**
T_4_	106	138.00 (120.00 to 168.00)	23.37 (21.81 to 24.76)	0.280 (0.082 to 0.458)	**0.004**	0.269 (0.088 to 0.448)	**0.006**
T_5_	110	145.00 (120.00 to 178.00)	23.55 (22.01 to 25.16)	0.288 (0.100 to 0.447)	**0.002**	0.350 (0.163 to 0.504)	**<0.001**
**PM**							
T_1_	49	130.00 (120.00 to 151.00)	23.63 (21.96 to 25.17)	0.311 (0.055 to 0.569)	**0.029**	0.102 (−0.205 to 0.358)	0.495
T_2_	47	134.00 (124.50 to 159.00)	24.17 (22.27 to 25.45)	0.185 (−0.113 to 0.454)	0.213	0.095 (−0.214 to 0.371)	0.534
T_3_	50	139.50 (127.50 to 168.00)	24.63 (22.44 to 25.99)	0.218 (−0.046 to 0.473)	0.129	0.170 (−0.139 to 0.427)	0.248
T_4_	49	145.00 (131.00 to 175.00)	24.89 (22.56 to 26.39)	0.183 (−0.118 to 0.427)	0.208	0.132 (−0.133 to 0.374)	0.376
T_5_	50	154.00 (138.00 to 180.00)	25.24 (22.70 to 26.62)	0.382 (−0.156 to 0.402)	0.126	0.170 (−0.106 to 0.447)	0.247
**NDM**							
*t*_−4_	73	125.00 (111.00 to 140.00)	22.86 (20.98 to 24.35)	0.104 (−0.132 to 0.355)	0.383	−0.043 (−0.293 to 0.197)	0.720
*t*_−3_	132	128.00 (110.00 to 147.00)	23.00 (21.01 to 24.64)	0.162 (−0.040 to 0.341)	0.064	0.015 (−0.159 to 0.178)	0.861
*t*_−2_	178	130.00 (109.00 to 152.00)	23.19 (21.15 to 24.96)	0.205 (0.042 to 0.355)	**0.006**	0.019 (−0.137 to 0.168)	0.802
*t*_−1_	215	135.00 (110.00 to 160.00)	23.46 (21.41 to 25.36)	0.230 (0.092 to 0.373)	**0.001**	0.034 (−0.102 to 0.172)	0.622
*t*_0_	221	142.00 (120.00 to 171.00)	24.02 (21.67 to 25.88)	0.199 (0.048 to 0.342)	**0.003**	0.105 (−0.036 to 0.227)	0.123
*t*_1_	150	144.00 (122.50 to 168.00)	24.42 (21.93 to 26.37)	0.214 (0.044 to 0.378)	**0.008**	0.223 (0.064 to 0.379)	**0.006**
*t*_2_	86	148.00 (125.00 to 170.00)	24.82 (22.38 to 26.41)	0.343 (0.155 to 0.505)	**0.001**	0.327 (0.134 to 0.507)	**0.002**
*t*_3_	33	151.00 (139.00 to 166.00)	24.95 (22.80 to 26.56)	0.419 (0.145 to 0.635)	**0.015**	0.468 (0.103 to 0.710)	**0.008**

*AL, axial length; H, height; PE, persistent emmetropia; PM, persistent myopia; NDM, newly developed myopia*;

* *correlation coefficients adjusted for age and sex. Bold font indicates to point the P values < 0.05*.

**Table 4:** Correlation between height and refraction in all participants.

**Time**	***n***	***H*** **(cm)**	**SE (D)**	***R*** **(95% CI)**	***P***	Adj. R^*^(95% CI)	***P***
**All**							
T_1_	413	125.00 (108.00 to 151.00)	0.00 (-4.13 to 9.50)	−0.162 (−0.256 to −0.062)	**0.001**	−0.120 (−0.214 to −0.025)	**0.015**
T_2_	399	130.00 (110.00 to 159.00)	0.00 (-5.00 to 9.00)	−0.207 (−0.296 to −0.109)	**<0.001**	−0.108 (−0.207 to 0.003)	**0.031**
T_3_	409	135.00 (120.00 to 168.00)	0.00 (-6.13 to 8.25)	−0.206 (−0.303 to −0.102)	**<0.001**	−0.098 (−0.190 to 0.001)	**0.048**
T_4_	406	140.75 (120.00 to 175.00)	−0.38 (-7.25 to 7.63)	−0.155 (−0.259 to −0.056)	**<0.001**	−0.274 (−0.363 to −0.187)	**0.002**
T_5_	414	150.00 (120.00 to 180.00)	−1.25 (-7.88 to 6.88)	−0.236 (−0.327 to −0.141)	**<0.001**	−0.144 (−0.251 to −0.040)	**0.003**

*H, height; SE, spherical equivalent*;

* *correlation coefficients adjusted for age and sex. Bold font indicates to point the P values < 0.05*.

During the five-year follow-up, on average, the children grew by 23.60 ± 4.65 cm in height, their AL increased by 1.39 ± 0.53 mm, and their SE change was −1.69 ± 1.29 D. The prevalence of myopia in each examination (from T_1_ to T_5_) was 12.1, 20.0, 32.9, 48.8, and 66.7%, respectively.

### Correlation of Height With Axial Length and Refraction

In each examination of all children, height and AL were positively and significantly correlated. The correlation was still statistically significant after adjusting for age and sex (Table 3). Conversely, height and refraction were negatively correlated before and after controlling for age and sex (Table 4).

**Figure 1:**
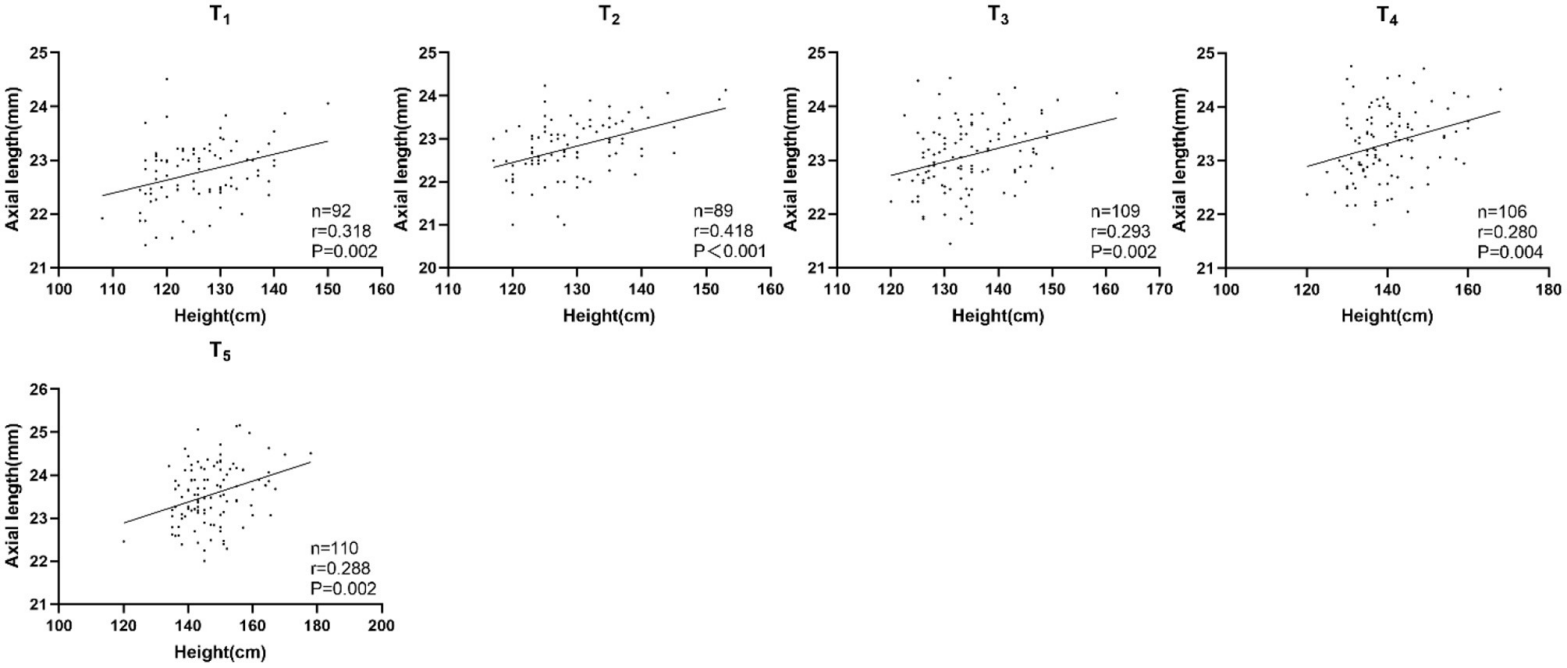
Correlation between height and axial length in the persistent emmetropia group.

In the persistent emmetropia group, height and AL were positively correlated in each examination. The correlation was statistically significant with or without adjusting for sex and age (Table 3; Figure 1).

In the persistent myopia group, the correlation between height and AL only existed at T_1_, and was no longer present after correcting for age and sex (Table 3).

**Figure 2:**
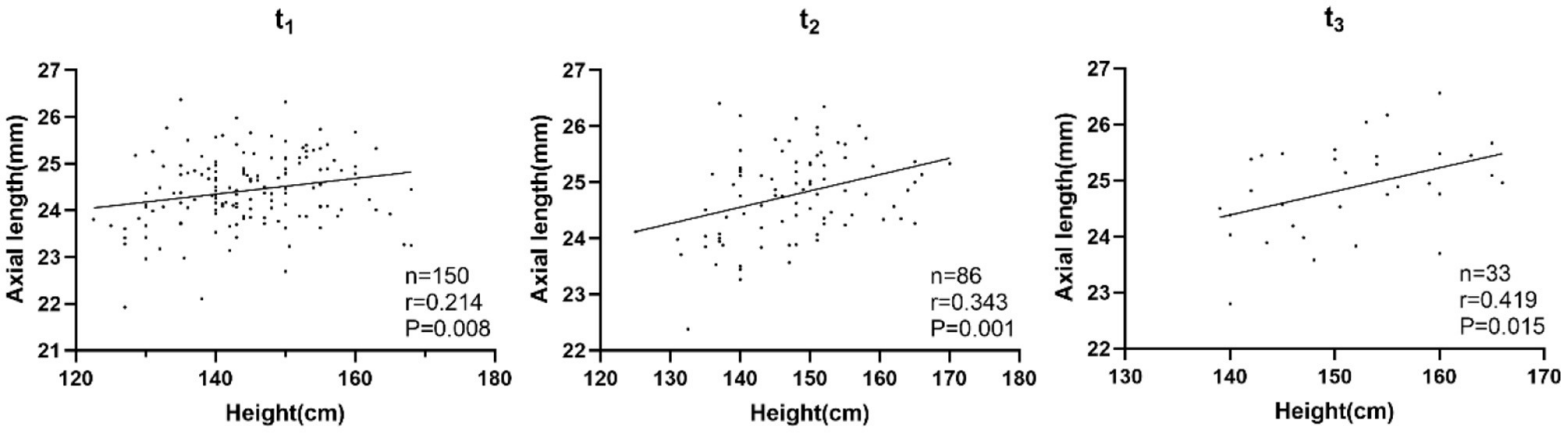
Correlation between height and axial length in the newly developed myopia group.

In the newly developed myopia group, height and AL were positively correlated at *t*_−2_, *t*_−1_, and *t*_0_ only before adjusting for confounding factors. From *t*_1_ to *t*_3_, height and AL were positively correlated both before and after controlling for sex and age (Table 3; Figure 2).

### Correlation Between Changes in Height and Changes in Axial Length

**Figure 3:**
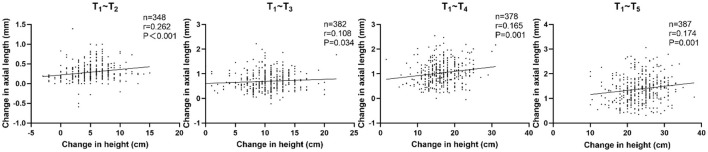
Correlation between changes in height and changes in axial length in all participants.

Correlations between the changes in height and AL were statistically significant for T_1_~T_2_, T_1_~T_3_, T_1_~T_4_, and T_1_~T_5_ (*r* = 0.262, *P* < 0.001; *r* = 0.108, *P* = 0.034; *r* = 0.165, *P* = 0.001; *r* = 0.174, *P* = 0.001, respectively). Furthermore, the correlations were still statistically significant for T_1_~T_2_, T_1_~T_4_, and T_1_~T_5_ after adjusting for age and sex (*r* = 0.187, *P* < 0.001; *r* = 0.154, *P* = 0.003; *r* = 0.154, *P* = 0.002; Table 5; Figure 3).

**Table 5:** Correlation between changes in height and changes in AL in different groups.

**Time**	***n***	**Δ***H* **(cm)**	**ΔAL (mm)**	***R*** **(95% CI)**	***P***	Adj. R^*^(95% CI)	***P***
**All**							
T_1_~T_2_	348	5.00 (−3.00 to 15.00)	0.25(−0.59 to 1.39)	0.262 (0.163 to 0.358)	**<0.001**	0.187 (0.086 to 0.288)	**<0.001**
T_1_~T_3_	382	10.00 (0.00 to 22.00)	0.62(−0.22 to 2.22)	0.108 (0.013 to 0.202)	**0.034**	0.068 (−0.035 to 0.170)	0.186
T_1_~T_4_	378	16.00(1.50 to 31.00)	1.00 (−0.05 to 2.54)	0.165 (0.065 to 0.263)	**0.001**	0.154 (0.047 to 0.258)	**0.003**
T_1_-~T_5_	387	24.00 (10.00 to 38.00)	1.37 (0.31 to 3.06)	0.174 (0.079 to 0.269)	**0.001**	0.154 (0.057 to 0.248)	**0.002**
**PE**							
T_1_~T_2_	79	4.00 (−2.00 to 13.00)	0.17 (−0.28 to 1.39)	0.198 (−0.220 to 0.407)	0.08	0.058 (−0.110 to 0.314)	0.615
T_1_~T_3_	91	9.50 (1.00 to 17.00)	0.41 (0.02 to 1.73)	−0.075 (−0.288 to 0.128)	0.477	−0.033 (−0.210 to 0.133)	0.757
T_1_~T_4_	88	14.50 (5.00 to 23.00)	0.64 (−0.05 to 2.08)	0.035 (−0.184 to 0.252)	0.748	−0.024 (−0.214 to 0.199)	0.827
T_1_~T_5_	92	22.50 (10.00 to 32.00)	0.88 (0.31 to 2.65)	0.120 (−0.103 to 0.334)	0.254	0.151 (−0.035 to 0.335)	0.156
**PM**							
T_1_~T_2_	46	5.00 (−1.00 to 10.00)	0.43 (0.00 to 0.82)	0.389 (0.094 to 0.623)	**0.008**	0.294 (−0.018 to 0.565)	0.053
T_1_~T_3_	49	11.00 (1.00 to 22.00)	0.85 (0.17 to 1.85)	0.234 (−0.092 to 0.520)	0.105	0.259 (−0.042 to 0.524)	0.079
T_1_~T_4_	48	18.00 (10.00 to 31.00)	1.17 (0.29 to 2.47)	0.113 (−0.232 to 0.444)	0.443	0.183 (−0.152 to 0.462)	0.223
T_1_~T_5_	49	25.00 (10.00 to 34.00)	1.56 (0.38 to 3.06)	−0.074 (−0.326 to 0.196)	0.612	0.036 (−0.243 to 0.317)	0.809
**NDM**							
*t*_−4_~*t*_0_	73	25.00 (15.00 to 32.00)	1.33 (0.42 to 2.61)	0.116 (−0.134 to 0.374)	0.328	0.054 (−0.199 to 0.363)	0.654
*t*_−3_~*t*_0_	132	17.50 (6.50 to 31.00)	1.14 (0.00 to 2.61)	−0.095 (−0.274 to 0.073)	0.277	−0.120 (−0.332 to 0.105)	0.173
*t*_−2_~*t*_0_	177	12.00 (1.00 to 24.00)	0.90 (0.00 to 1.99)	−0.038 (−0.182 to 0.107)	0.620	0.115 (−0.035 to 0.259)	0.130
*t*_−1_~*t*_0_	211	6.00 (−2.00 to 16.00)	0.50 (−1.58 to 1.47)	0.018 (−0.127 to 0.159)	0.795	0.037 (−0.073 to 0.167)	0.596
*t*_0_~*t*_1_	145	6.00 (−1.00 to 14.00)	0.43 (−0.35 to 2.41)	0.031 (−0.118 to 0.192)	0.712	0.054 (−0.060 to 0.186)	0.524
*t*_0_~*t*_2_	81	13.00 (5.00 to 23.00)	0.80 (−0.02 to 2.00)	0.289 (0.084 to 0.480)	**0.009**	0.317 (0.126 to 0.507)	**0.004**
*t*_0_~*t*_3_	29	19.00 (8.50 to 26.00)	1.13 (0.50 to 2.29)	0.362 (−0.056 to 0.642)	0.054	0.278 (−0.122 to 0.610)	0.161

*AL, axial length; H, height; PE, persistent emmetropia; PM, persistent myopia; NDM, newly developed myopia*;

* *correlation coefficients adjusted for age and sex. Bold font indicates to point the P values < 0.05*.

**Figure 4:**
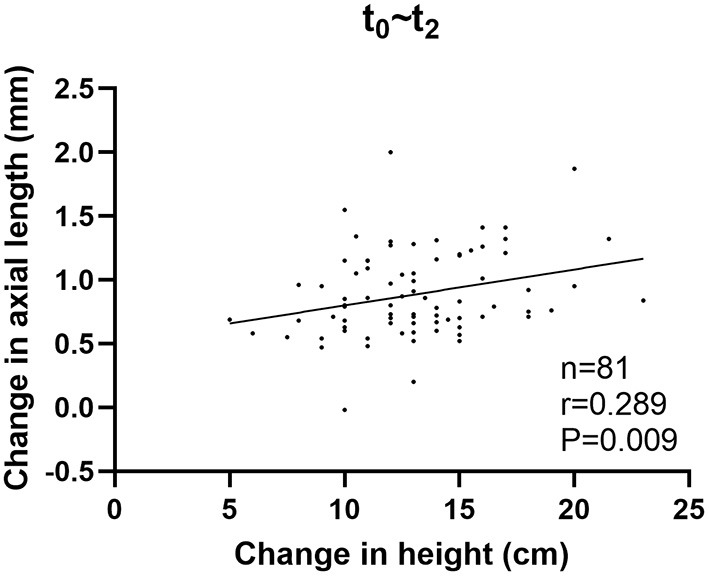
Correlation between changes in height and changes in axial length in the newly developed myopia group after the onset of myopia.

Significant correlations were found in the newly developed myopia group after the onset of myopia. Changes in height and AL were positively correlated both before and after correcting for age and sex for *t*_0_~*t*_2_ (*r* = 0.289, *P* = 0.009; *r* = 0.317, *P* = 0.004), while no significant correlations were found before myopia onset (Table 5; Figure 4).

No significant correlation was observed between changes in AL and changes in height in the persistent emmetropia group. Similarly, no significant associations were found in the persistent myopia group, except for T_1_~T_2_ (*r* = 0.388, *P* = 0.008) before adjusting for the confounding factors (Table 5).

### Correlation Between Changes in Height and Refraction

Table 6 shows the correlation between the changes in height and refraction. Changes in height and SE were negatively correlated for T_1_~T_3_, T_1_~T_4_, and T_1_~T_5_ (*r* = −0.097, *P* = 0.049; *r* = −0.186, *P* < 0.001; *r* = −0.167, *P* = 0.001). The pattern of correlation in the sub-groups was similar to that of changes in AL and height, but with negative correlation coefficients.

**Table 6:** Correlation between changes in height and changes in refraction in different groups.

**Time**	***n***	**Δ*H* (cm)**	**ΔSE (D)**	***R*** **(95% CI)**	***P***	Adj. R^*^(95% CI)	***P***
**All**							
T_1_~T_2_	398	5.00 (−3.00 to 15.00)	−0.25 (−2.25 to 2.50)	−0.057 (−0.161 to 0.041)	0.26	−0.092 (−0.182 to 0.004)	0.067
T_1_~T_3_	408	10.00 (0.00 to 22.00)	−0.50 (−3.50 to 2.00)	−0.097 (−0.188 to 0.000)	**0.049**	−0.082 (−0.173 to 0.020)	0.100
T_1_~T_4_	405	16.00 (1.50 to 31.00)	−0.90 (-5.30 to 1.80)	−0.186 (−0.285 to −0.086)	**<0.001**	−0.154 (−0.258 to −0.057)	**0.002**
T_1_~T_5_	413	24.00 (10.00 to 38.00)	−1.50 (-6.00 to 1.75)	−0.167 (−0.262 to −0.072)	**0.001**	−0.131 (−0.221 to −0.037)	**0.008**
**PE**							
T_1_~T_2_	104	4.00 (−2.00 to 13.00)	0 (−0.75 to 0.75)	−0.021 (−0.211 to 0.164)	0.832	−0.037 (−0.229 to 0.146)	0.709
T_1_~T_3_	110	9.00 (1.00 to 17.00)	0 (−0.75 to 0.75)	0.056 (−0.126 to 0.238)	0.563	0.052 (−0.114 to 0.231)	0.592
T_1_~T_4_	106	15.00 (5.00 to 23.00)	0 (−0.88 to 0.75)	0.008 (−0.184 to 0.185)	0.936	−0.009 (−0.198 to 0.182)	0.925
T_1_~T_5_	110	22.00(10.00 to 32.00)	−0.25 (−1.00 to 0.75)	−0.039 (−0.228 to 0.146)	0.683	−0.041 (−0.209 to 0.147)	0.676
**PM**							
T_1_~T_2_	47	5.00 (−1.00 to 10.00)	−0.75 (−2.00 to 0.63)	−0.264 (−0.558 to 0.018)	0.073	−0.199 (−0.484 to 0.108)	0.191
T_1_~T_3_	48	11.00 (1.00 to 22.00)	−1.75 (−3.50 to 0.13)	−0.196 (−0.459 to 0.094)	0.181	−0.150 (−0.401 to 0.105)	0.321
T_1_~T_4_	48	18.00 (10.00 to 31.00)	−2.30 (-5.30 to 0.10)	−0.141 (−0.460 to 0.169)	0.338	−0.127 (−0.412 to 0.181)	0.401
T_1_~T_5_	49	25.00 (10.00 to 34.00)	−3.00 (-6.00 to −0.13)	0.057 (−0.225 to 0.293)	0.695	0.036 (−0.266 to 0.331)	0.809
**NDM**							
*t*_−4_~*t*_0_	74	24.75 (15.00 to 32.00)	−1.25 (−3.63 to −0.25)	0.051 (−0.205 to 0.283)	0.666	0.014 (−0.174 to 0.237)	0.905
*t*_−3_~*t*_0_	138	18.00 (6.50 to 31.00)	−1.25 (−2.75 to −0.25)	0.019 (−0.149 to 0.186)	0.824	0.050 (−0.130 to 0.213)	0.564
*t*_−2_~*t*_0_	188	12.00 (1.00 to 24.00)	−1.00 (−2.75 to −0.25)	0.137 (−0.007 to 0.276)	0.061	0.073 (−0.075 to 0.213)	0.321
*t*_−1_~*t*_0_	221	6.00 (−2.00 to 16.00)	−0.75 (−2.38 to −0.13)	0.001 (−0.137 to 0.130)	0.991	−0.003 (−0.142 to 0.138)	0.969
*t*_0_~*t*_1_	148	6.00 (−1.00 to 14.00)	−0.75 (−2.25 to 0.75)	−0.016 (−0.177 to 0.151)	0.850	−0.063 (−0.205 to 0.067)	0.451
*t*_0_~*t*_2_	84	13.00 (5.00 to 23.00)	−1.50 (−3.25 to 0.75)	−0.221 (−0.391 to −0.037)	**0.044**	−0.219 (−0.391 to −0.055)	**0.048**
*t*_0_~*t*_3_	32	19.00 (8.50 to 28.00)	−1.94 (-4.00 to 0.75)	−0.356 (−0.590 to −0.044)	**0.046**	−0.291 (−0.544 to 0.011)	0.119

*SE, spherical equivalent; H, height; PE, persistent emmetropia; PM, persistent myopia; NDM, newly developed myopia*;

* *correlation coefficients adjusted for age and sex. Bold font indicates to point the P values < 0.05*.

## Discussion

This cohort study was conducted in Jinhua, a city located in eastern China, where the incidence of myopia is relatively high (28). In total, 414 children aged 6–9 participated in the study and completed a five-year series of follow-up examinations from 2015 to 2020, in which every child was examined every 12 months. A correlation was found between the growth in height and the increase in AL in children and adolescents, especially in the newly developed myopia group. Our results suggest that children may also experience increased AL growth when they present with rapid height growth.

We assessed the relationship between the changes in height and the increase in AL and found that they were positively correlated in children aged 6–9. This is essentially consistent with the results of Huang et al.'s study (19), which included 65 children aged 7–9 years old followed up every 6 months in a three-year period. They concluded that growth in height and AL during the research period were correlated. Compared with their study, the present one included more participants and had longer follow-up. Furthermore, we proved that the correlation existed not only in the whole period (T_1_~T_5_), but also at every follow-up time point (T_1_~T_2_, T_1_~T_3_, T_1_~T_4_, T_1_~T_5_).

However, the study by Li et al. did not find that the changes in height and AL were correlated (21). In their study, a total of 452 children aged 6–8 years accepted measurements every year during the 3-year follow-up period. They analyzed the relationship between the mean change in AL and the mean change in height through multivariate linear regression analysis, finding that they were not correlated at any point in the 3-year follow-up period (2015–2014, 2016–2014, or 2017–2014). This may be related to the lack of representativeness of the sample, composed of students of grades 1 and 2 from a single school. It may also be related with the shorter follow-up time (3 years) and the fact that refractive status was not considered. Kearney et al. (20) concluded that changes in height and AL were correlated in the persistent emmetropia group (*n* = 55), but not in the newly developed myopia group, in 105 subjects aged 5–20 years, with examination conducted every 2 years for 4 years. The disagreement between the results of the study by Kearney et al. and ours may result from the difference in the age of the participants and the follow-up time intervals.

We also found a positive correlation between changes in height and AL after myopia onset in the newly developed myopia group. That might be due to the fact that children have a peak incidence of myopia at the age of growth spurt at least in Chinese. A previous study reported that the onset of myopia and the peak of its progression may be associated with growth spurts (16). Moreover, AL elongation and growth in height may be partially mediated by the same genes (18). The changes in height are the result of both genetic and environmental factors (29, 30), and the same applies to AL (31, 32). The experiment by He proved that the correlation between AL and height is largely (89%) attributable to shared genes (18). In addition to genes, hormones play an important role: many hormones involved in height growth, such as GH, IGF-1, and TH, have also been shown to accelerate the growth of eyes (33–36). Although there may be shared genes and hormones related to both growths, height is more susceptible to nutritional environmental factors and gastrointestinal infection, while AL growth is more susceptible of being modified by illumination and visual cues.

In the present study, we also explored the relationship between height and AL for different refractive status. Selovic et al. found that height and AL were positively correlated in persistent emmetropes by analyzing the data of 1,600 pupils (37). However, they neither investigated the correlation in newly developed or persistent myopes, nor conducted a follow-up study. Our further exploration also revealed that height and AL were positively correlated in every examination in the persistent emmetropia group as well as in the newly developed myopia group after the onset of myopia. However, the association did not exist in any examination in the newly developed myopia group before the onset of myopia or in the persistent myopia group. Whether the onset of myopia plays a role in the relationship between height and AL in children and adolescents, further researches are required.

Similar results were obtained for the correlation between height and refraction, because refraction is largely determined by the AL (38, 39). Though AL plays an important role in refraction, a longer AL doesn't necessarily mean more myopic. Emmetropia is a balance between AL, corneal power and lens power (40). That means longer eyes can be compensated by less lens power or flatter corneas to keep emmetropic (41–45). So future studies should take lens power and corneal radius of curvature into consideration to account for their possible compensation of greater axial growth.

The current research has proved that changes in height and AL are positively correlated in the transition from childhood to adolescence. Myopia gradually becomes prevalent from the age of 6–9 years old (6, 46–49), when height also grows relatively fast (50). Yip et al. (16) argued that children who experienced peak height velocity earlier may also become myopic earlier. Our study further found that when children grow fast in height, their AL may also elongate quickly, just at the time they may be more likely to become myopic in this environment. Thus, observing the growth rate of children can serve as an indicator to monitor the growth velocity of their AL. When a child is in a stage of rapid height growth, we may need to be aware that his/her AL is also in a period of easy elongation. The elongation of the AL is closely related to the occurrence and development of myopia. As to whether the strengthening of myopia prevention and control measures can slow the growth of AL in the period of rapid height growth, further research is warranted.

Based on prior studies, we further proved the correlation between changes in height and changes in AL. However, there are some limitations to our study. First, we did not produce genetic data or measure hormone levels. Therefore, we cannot directly prove whether the correlation between height and AL is mediated by genes or hormones. Second, we did not use a questionnaire to acquire information that may be associated with the onset of myopia, such as reading and writing distance, and time spent outdoors. Such information will help us better understand the development of myopia in our participants. Third, some of the subjects in our study may have grown into adolescence later in the follow-up period. Adolescents are likely to grow faster than children (51). However, we didn't have the exact puberty parameters such as age of maximum height velocity, age of menarche and voice changes. Although, in agreement with Yip et al. (16), we could observe a correlation between the time of rapid growth of AL and height, to adjust for antecedents of the pubertal peak, detailed information of puberty and a longer follow up time would be required. Finally, the follow up time is still too short to include the whole period of accelerated growth. We chose children aged 6–9 years old and followed up for 5 years which covers the time of rapid change of refraction based on our previous study (52). However, some subjects may be outside the peak of accelerated growth. A longer follow up period would be necessary to clarify the relationship between the peak of accelerated growth and the progression of myopia, and changes in AL and height after the onset of myopia in future studies.

Ulaganathan et al. (53) previously described that the mean amplitude of daily variations in AL is 0.029± 0.007 mm, thus, minor variations could be neglected when considering yearly AL changes. So, the AL was not measured at the same time each day in this study. The non-cycloplegic refraction may render an overestimation of myopia. However, it may have less effect in a longitudinal study such as our own, which monitored the progression of refraction in the same population (6).

In summary, we suggest that during the growth of school-age children, a significant correlation exists not only between AL and height, but also between AL growth and height growth, especially in children with newly developed myopia. This indicates that during the period of rapid height growth, the elongation of AL also needs to be considered. Whether the strengthening of outdoor activities or other myopia control measures can delay the elongation of AL during the rapid height growth period may be an urgent question that needs to be answered.

## Data Availability Statement

The raw data supporting the conclusions of this article will be made available by the authors, without undue reservation.

## Ethics Statement

The studies involving human participants were reviewed and approved by the Ethics Committee of Jinhua Eye Hospital. Written informed consent to participate in this study was provided by the participants' legal guardian/next of kin.

## Author Contributions

LT: research design and collect data. CW, YP, MX, and MW: technical assistance and guidance. XY: research and academic guidance. All authors contributed to the article and approved the submitted version.

## Funding

This work was supported by the National Natural Science Foundation of China Grant 82070995.

## Conflict of Interest

The authors declare that the research was conducted in the absence of any commercial or financial relationships that could be construed as a potential conflict of interest.

## Publisher's Note

All claims expressed in this article are solely those of the authors and do not necessarily represent those of their affiliated organizations, or those of the publisher, the editors and the reviewers. Any product that may be evaluated in this article, or claim that may be made by its manufacturer, is not guaranteed or endorsed by the publisher.
